# Intense Brilliancy of Lightning

**Published:** 1891-12

**Authors:** 


### THE INTENSE BRILLIANCY OF LIGHTNING.

One consequence of the short duration of lightning is an apparent diminution of its brilliancy. It has been proven that light cannot produce its full effect on the eye unless it remains at least as long as one-tenth of a second; but lightning lasts only the ten-thousandth part of a second, and it follows from this that what we see is one hundred thousand times less bright than it really is. When we recollect that even thus diminished its brilliancy is such as to cause temporary blindness if too closely watched, we may feel grateful that we cannot see it in its true vividness, for our human powers of vision would be too weak to bear such a sudden and overwhelming illumination.—GAILLARD'S *Electricity*.

